# A Comparative Study on the Mechanical, Thermal and Morphological Characterization of Poly(lactic acid)/Epoxidized Palm Oil Blend

**DOI:** 10.3390/ijms13055878

**Published:** 2012-05-16

**Authors:** V. S. Giita Silverajah, Nor Azowa Ibrahim, Wan Md Zin Wan Yunus, Hazimah Abu Hassan, Chieng Buong Woei

**Affiliations:** 1 Department of Chemistry, Faculty of Science, University Putra Malaysia, UPM Serdang, Selangor 43400, Malaysia; E-Mail: chieng891@hotmail.com; 2 Chemistry Department, Center for Defence Foundation Studies, National Defence University of Malaysia, Kuala Lumpur 57000, Malaysia; E-Mail: wanmdzin@upnm.edu.my; 3 Advanced Oleochemical Technology Division, Malaysian Palm Oil Board, Bandar Baru Bangi 43650, Malaysia; E-Mail: hazimah@mpob.gov.my

**Keywords:** melt blending, plasticizer, bioplastic, biodegradable plastic

## Abstract

In this work, poly(lactic acid) (PLA) a fully biodegradable thermoplastic polymer matrix was melt blended with three different epoxidized palm oil (EPO). The aim of this research was to enhance the flexibility, mechanical and thermal properties of PLA. The blends were prepared at various EPO contents of 1, 2, 3, 4 and 5 wt% and characterized. The SEM analysis evidenced successful modification on the neat PLA brittle morphology. Tensile tests indicate that the addition of 1 wt% EPO is sufficient to improve the strength and flexibility compared to neat PLA. Additionally, the flexural and impact properties were also enhanced. Further, DSC analysis showed that the addition of EPO results in a decrease in T_g_, which implies an increase in the PLA chain mobility. In the presence of 1 wt% EPO, TGA results revealed significant increase in the thermal stability by 27%. Among the three EPOs used, EPO(3) showed the best mechanical and thermal properties compared to the other EPO’s, with an optimum loading of 1 wt%. Conclusively, EPO showed a promising outcome to overcome the brittleness and improve the overall properties of neat PLA, thus can be considered as a potential plasticizer.

## 1. Introduction

Over the past few decades, most composites used are petrochemically derived products such as polyethylene, polypropylene, and polyethylene terephthalate (PET) that are non-biodegradable. With the growing concern about environmental pollution, the accumulation of plastic waste in landfills needs immediate resolution. In addition, increasing global environmental problems such as greenhouse gas emission and diminishing fossil resources have also focused attention on the development of degradable plastics [1]. Biodegradable plastics offer a solution in managing packaging waste. Biodegradable plastics are receiving much interest in the emerging topic of green chemistry since they are environment-friendly, compostable and biodegradable, as well as being obtained from renewable and sustainable resources. Moreover, they reduce our dependency on depleting fossil fuels and reduce the generation of hazardous substances [2].

The widely used biodegradable polymers are aliphatic polyesters and proteins, such as poly(lactic acid) (PLA), poly(3-hydroxybutyrate) (PHB), poly(ɛ-caprolactone) (PCL), and starch [3]. One of the most promising and attractive thermoplastic materials being developed is PLA. PLA is obtained from glucose fermentation of naturally occurring biomass such as corn, potato, sugar beet, and sugar cane [4,5]. Commercially, PLAs can be synthesized by the condensation polymerization of lactic acid (2-hydroxy propionic acid) or ring-opening polymerization of the cyclic lactide dimer (lactide monomer) [6].

PLA, a linear aliphatic polyester, is considered as the most innovative alternatives for conventional petroleum-based polymers because it has similar mechanical properties such as density, glass transition temperature (T_g_), tensile strength, and Young’s modulus as to those of PET [7]. Furthermore, PLA has good potential due to its excellent properties, such as good mechanical properties (especially in strength and modulus), easy processability, excellent degradability and transparency [3]. However, the main drawbacks of PLA are due to its high brittleness, low toughness and low tensile elongation [8].

Therefore, considerable efforts have been made to enhance the characteristics of PLA by employing plasticizer. Ali *et al*. [9] investigated the effect of epoxidized soybean oil (ESO) on PLA. They revealed that this blend lowered the T_g_ and dynamic storage modulus, while increased the ability of PLA to cold crystallization and elongation-at-break compared with neat PLA. Another study by Emad *et al*. [10] on the effect of epoxidized palm oil (EPO) on PLA prepared by solution casting process showed high thermal stability and improvement of mechanical properties. PLA plasticization can also be achieved by blending with commercial or synthesized plasticizers such as poly(ethylene glycol), glycerol, citrate ester, starch, and oligomeric lactic acid [11]. The choice of plasticizers employed is restricted by its biodegradability, non-toxicity, low migration rates and good miscibility with PLA matrix by producing a homogeneous blend [12].

Besides that, if the films are intended to be applied for food packaging, the method used in the preparation has to meet the regulations governing the use of chemicals in food-contact applications. Blending is an economic technique for producing new polymer materials at low cost and desirable properties combination for specific end use. Several approaches have been proposed in the blend preparation. These approaches can be classified into two groups: (i) wet-based blending, *i.e.*, *in situ* polymerization of monomer with additive and solvent blending, and (ii) melt blending of polymer with additive. Each method has its advantages and limitations. Preparation of blends by using wet-based technique involves the dispersion of additive and polymer in water or organic solvents, in order to isolate the particles [13]. The advantages of this method include uniform thickness distribution, maximum optical purity and extremely low haze. However, this approach is time-consuming and not environmentally friendly as it involves solvent [14].

In preparing blends via a melt-blending (or dry-mixing) approach, the polymer and additives are first dry-mixed and then melt-blended by using conventional plastic processing equipment. The absence of solvent in this technique means this approach is more environmental friendly. Furthermore, the melt-blending technique is compatible with the existing processing equipment, such as extruders, injection molders, and mixing chambers (internal mixer), and thus is more effective in the mass production of blends [13]. Moreover, this method is more preferable in food-contact applications.

The present work is focused on plasticizing PLA with three different epoxidized palm oil (EPO) via melt blending technique. EPO is an epoxide derivative of glycerol ester mixture with various saturated and unsaturated fatty acids. In addition, EPO is a renewable, biodegradable, environmentally friendly and cheap raw material [10]. Thus, the aim of this study is to obtain films with enhanced mechanical and thermal properties that are able to be applied in packaging formulations.

## 2. Results and Discussion

### 2.1. Effect of EPO Loading on the Tensile Properties of Blends

**Figure 1:**
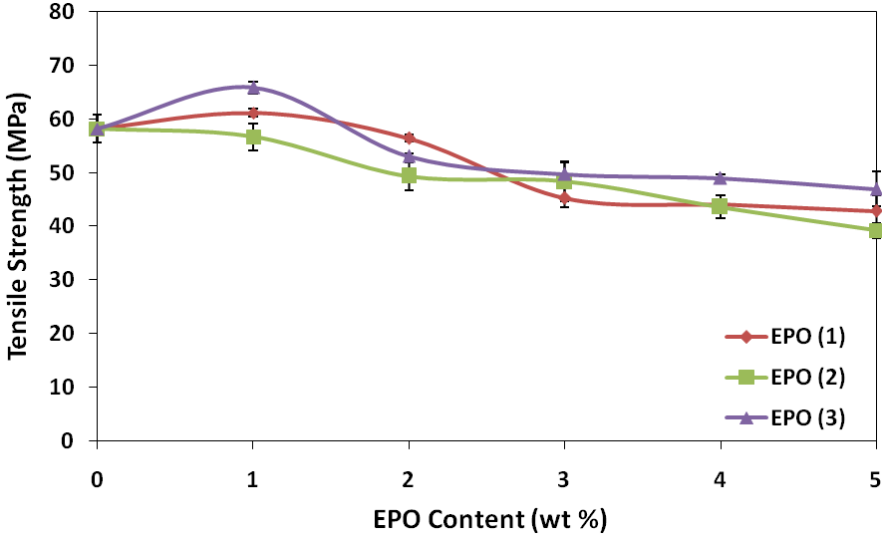
Effect of epoxidized palm oil (EPO) content on tensile strength of poly(lactic acid) (PLA)/EPO blends.

**Figure 2:**
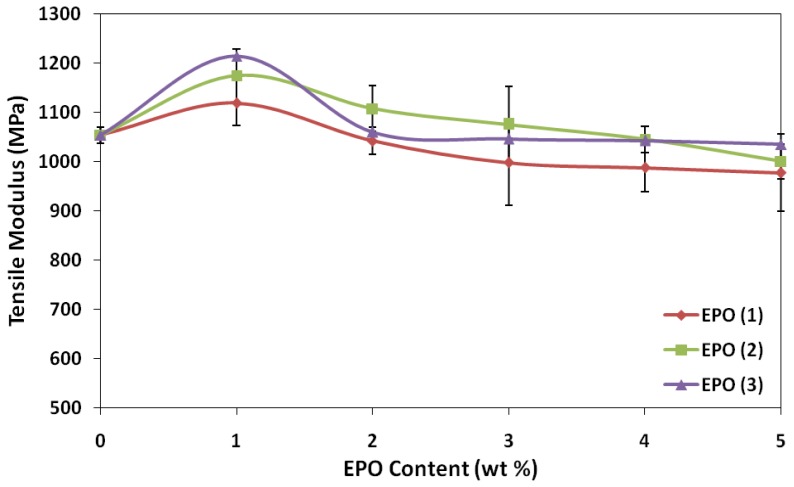
Effect of EPO content on tensile modulus of PLA/EPO blends.

**Figure 3:**
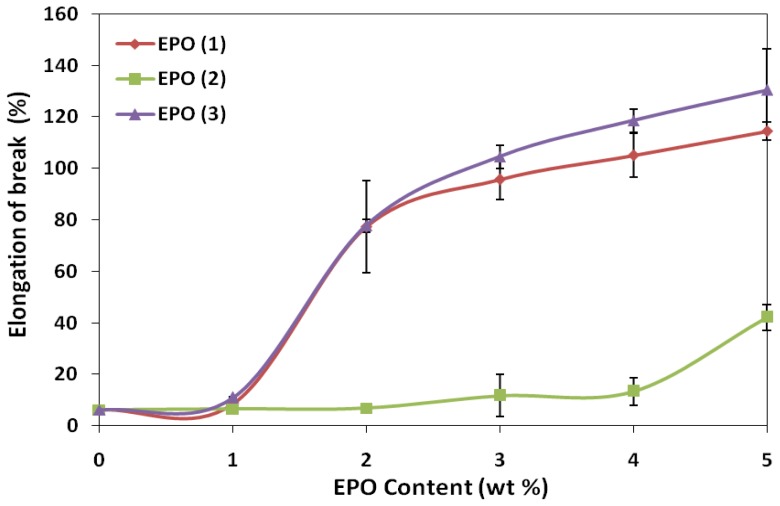
Effect of EPO content on elongation-at-break of PLA/EPO blends.

The tensile strength, elongation at break and tensile modulus were determined at room temperature (25 °C) to ascertain the films quality in a packaging use for instance. Results of the tensile measurements, including tensile strength, tensile modulus and elongation-at-break, are displayed graphically in Figures 1, 2 and 3. Addition of 1 wt% EPO significantly improved the tensile strength of PLA/EPO(1) and PLA/EPO(3) blends by approximately 5% and 13% respectively. This suggests that EPO acts as plasticizer, which increases the interaction at the phase boundaries and improves the flexibility of blends. Generally, the compounded EPO is dispersed in the interphase between the PLA chains. This results in strong interphase interaction, which reduces the stress concentration point when tensile load is applied on the blends and consequently produces higher mechanical strength [15,16].

However, a decrease by 2.6% in the tensile strength of PLA/1 wt% EPO(2) was observed. The tensile strength of blends decreased with the addition of EPO above 1 wt% in all the three types of EPO blends, indicating that there was no improvement in interaction between PLA and EPO. The drops in the tensile strength may be contributed by the plasticizer-plasticizer interaction which dominates at higher EPO content [17]. In addition, when the amount of EPO is above 1 wt%, only part of the EPO locates in the interfacial area, and the excess is dispersed in the matrix, affecting its homogeneity and consequently reducing the tensile strength of the blends [18]. Thus, only 1 wt% EPO is sufficient to enhance the tensile strength and PLA/1 wt% EPO(3) displayed the highest tensile strength of 65.8 MPa compared to neat PLA, 58.2 MPa.

Neat PLA exhibited a tensile modulus value of 1054 MPa, and attained the highest modulus with 1 wt% EPO for PLA/EPO(1) (1119 MPa), PLA/EPO(2) (1175 MPa) and PLA/EPO(3) (1214 MPa). According to Ibrahim *et al*. [17], a high tensile modulus signifies that the material is rigid, thus more stress is required to produce a given amount of strain, which means it resists deformation or stretch. EPO has been found to be efficient in stiffening PLA polymers as the tensile modulus increases by 6.2%, 11.5% and 15.2% for PLA/1 wt% EPO blend of EPO(1), EPO(2) and EPO(3) respectively. The increase in tensile modulus was attributed by blends having the stiffness of PLA and the polymer-plasticizer interaction which made the blends more rigid. Above 1 wt%, the tensile modulus decreased and plateaued. This could be considered the critical interfacial concentration, which is the minimum value of interfacial saturation for EPO in the interphase [19]. The trend of tensile modulus graph is similar to the tensile strength results.

Moreover, the elongation of PLA/EPO blends increases with the addition of plasticizer. The elongation at break of PLA/1 wt% EPO blends of EPO(1), EPO(2) and EPO(3) were 8.6%, 6.6% and 11.1% respectively, while neat PLA exhibited 6.3%. It has been observed that elongation at break of blends increases significantly with increasing EPO loading. As expected, PLA blends with 5 wt% EPO loading bear maximum elongation. With 5 wt% EPO loadings, PLA/EPO(1), PLA/EPO(2) and PLA/EPO(3) displayed elongation of 114%, 42% and 130% respectively. In general, plasticizer is introduced to a polymer matrix to overcome the film brittleness caused by extensive intermolecular interactions. Thus, the presence of plasticizer, EPO, reduces these intermolecular forces and increases the mobility of PLA chains, thereby enhancing the flexibility and extensibility of the PLA/EPO films. Consequently, higher EPO loading produces film with lower tensile strength but higher elongation [20].

Generally, incorporation of plasticizer results in lower tensile strength and modulus, but a contrary result was observed. The slightly higher tensile strength and modulus is contributed by the strength of interaction between polymer-plasticizer, PLA-EPO, which can be explained by the epoxy content also known as oxirane oxygen content (OOC) of the epoxidized oil. The OOC value indicates the epoxy groups present in the plasticizer, while the iodine value indicates the degree of unsaturation. An effective plasticizer must contain two types of structural components, which is polar and non-polar. The OOC represents the polar portion apart from carbonyl group of carboxylic ester functionality. Higher OOC of EPO(3) (3.58%) compared to EPO(1) (3.23%) and EPO(2) (1.75%) resembles stronger interaction (hydrogen bonds) between PLA and 1 wt% EPO(3) compared to another two blends. According to Wypych [17], polar groups in a plasticizer improve tensile strength, but flexibility is only moderately improved since there are points of high cohesion at many points along the chain. Polar groups in a plasticizer are essential for good compatibility, which can be observed from SEM micrograph of PLA/1 wt% EPO(3) showing good structural morphology. Eventually, if the plasticizer used is very non-polar (low OOC value but high iodine value 3.57%), it results in poor interaction and eventually incompatibility between polymer and plasticizer, as resembled by PLA and EPO(2).

### 2.2. Effect of EPO Loading on the Flexural Properties of Blends

**Figure 4:**
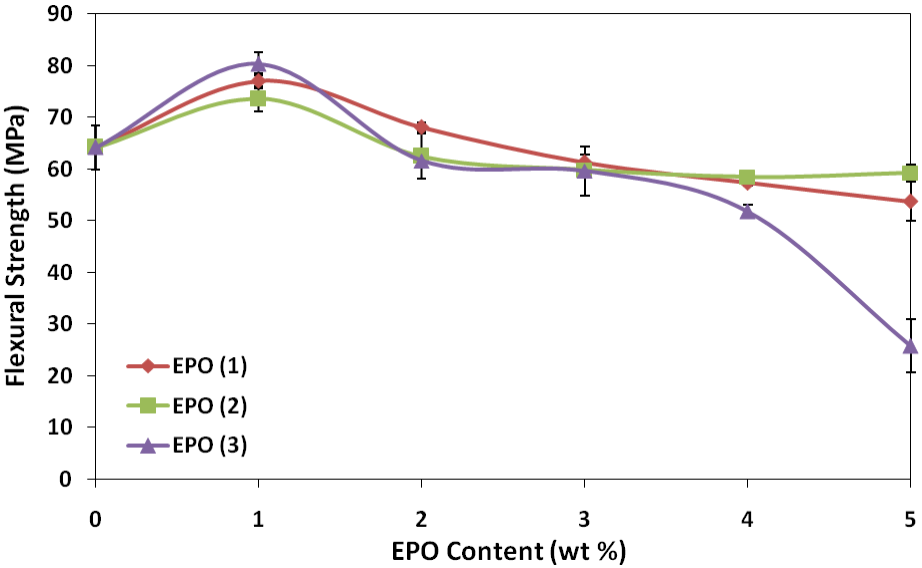
Effect of EPO content on flexural strength of PLA/EPO blends.

**Figure 5:**
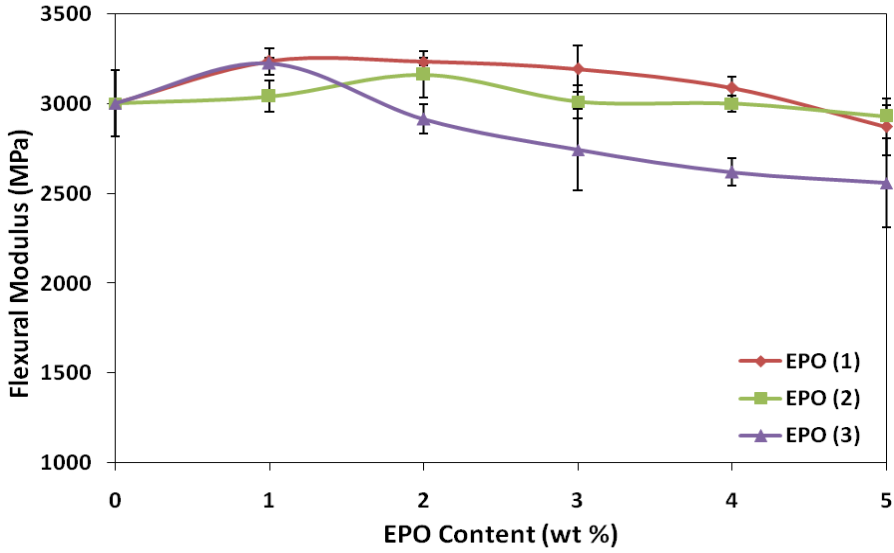
Effect of EPO content on flexural modulus of PLA/EPO blends.

Flexural strength reflects the ability of material to resist compression and tension stress simultaneously. The effect of EPO on the flexural strength and modulus of PLA/EPO blends are presented in Figures 4 and 5. The flexural strength of PLA enhanced significantly upon adding 1 wt% EPO into the matrix, from 64.2 MPa (neat PLA) to 77.0, 73.6 and 80.3 MPa, for PLA/EPO(1), PLA/EPO(2) and PLA/EPO(3). The trend of flexural strength for PLA/EPO(1) and PLA/EPO(2) blends are quite constant.

Conversely, the trend of flexural modulus for PLA/EPO(3) exhibited a maximum with 1 wt% and eventually a decrease. The high flexural strength was contributed by the improved adhesion between the PLA matrix and EPO, which provides an increase in stress transfer from the matrix to the plasticizer. Thus, this increases the stress at failure and contributes to the higher values for flexural strength [21]. However, further increase in the EPO loadings decreases the flexural strength indicating that presence of excess EPO reduces the stiffness of the PLA molecular chain. Moreover, increasing EPO content above 1 wt% adds to the occurrence of voids in the blends, which influence the local stress acting on the blends [22].

### 2.3. Effect of EPO Loading on the Impact Properties of Blends

**Figure 6:**
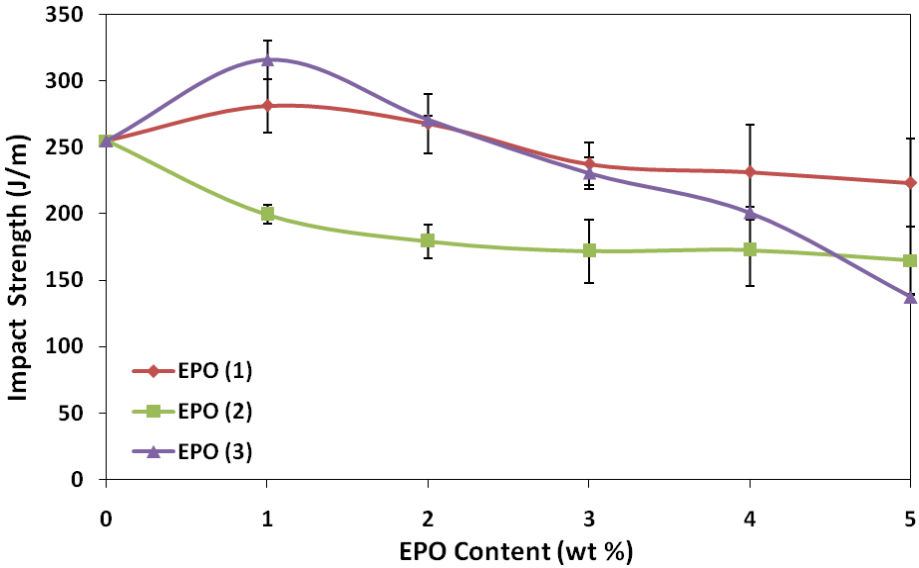
Effect of EPO content on impact strength of PLA/EPO blends.

Impact test reflects the ability of material absorbing energy at fracture, when exposed to sudden impact [23]. The impact properties of PLA/EPO blends are shown in Figure 6. The impact strength of PLA is improved by 10% (PLA/1 wt% EPO(1)) and 24% (PLA/1 wt% EPO(3)) from 254.8 J/m (neat PLA), with the presence of 1 wt% of EPO. These results indicate that reactions might occur between epoxy groups of EPO and hydroxyl groups at the terminals of the PLA matrix, improving the interfacial adhesion and leads to an increase in the impact strength. However, further addition of EPO in these blends beyond optimum amount (1 wt% EPO), the impact strength gradually decreased. In this case, excess epoxy groups from EPO contribute to higher plasticizer-plasticizer interaction, along with poor interaction between PLA and EPO. This triggers the formation of voids as observed in SEM analysis and eventually decreases the impact strength [24]. Conversely, the impact strength of PLA/EPO(2) blends decreased by approximately 20% and plateaued. This indicates poor interfacial adhesion in the blends, thus decreasing the toughness with increasing plasticizer content and finally remains constant at a particular point [21].

### 2.4. Morphological Study of PLA/EPO Blends

**Figure 7:**
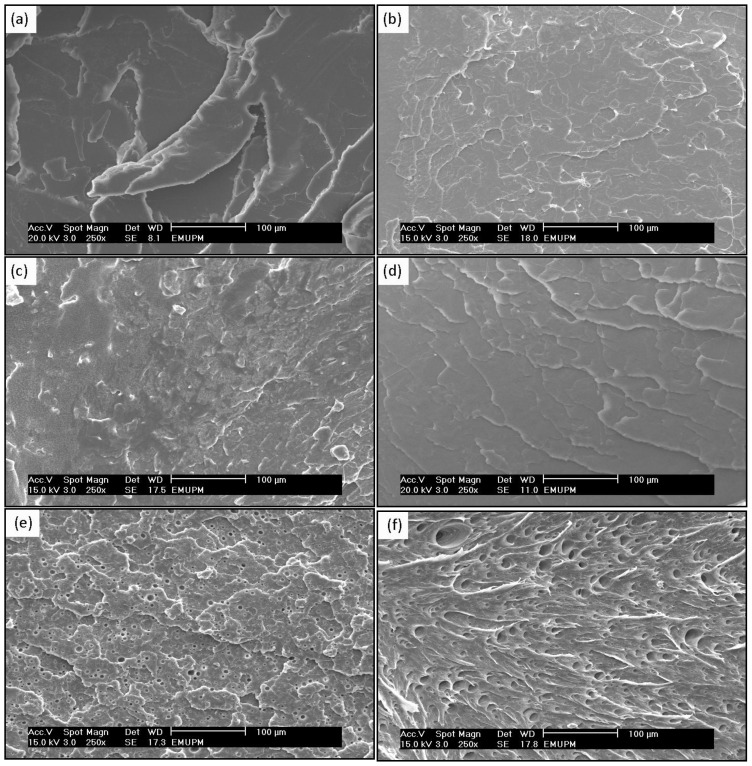
SEM images of (**a**) neat PLA; (**b**) PLA/1 wt% EPO(1); (**c**) PLA/1 wt% EPO(2); (**d**) PLA/1 wt% EPO(3); (**e**) PLA/5 wt% EPO (top view); and (**f**) PLA/5 wt% EPO (side view) blends.

Scanning electron microscopy (SEM) was used to observe the surface morphology of the fractured tensile specimens and qualitatively visualize the state of dispersion of the EPO in the polymer matrix. Figure 7 displays the surface micrographs of neat PLA and PLA/EPO blends. A typical tensile fracture surface of PLA is shown in Figure 7a, which exhibited crack surface corresponding to brittle fracture nature of PLA [25]. The presence of 1 wt% EPO significantly influences the morphological structural changes of the blend. As shown in Figure 7b–d, in the presence of the 1 wt% EPO, the deformation of PLA phase creates neither a brittle fracture, as observed in the neat PLA blend, nor defect cavities, as observed in the PLA/5 wt% EPO blends.

Figure 7b shows good compatible morphologies however there are presence of fibrils. Figure 7c shows evident signs of plasticization in the PLA matrix. However, the dispersion of EPO(2) in the PLA matrix is not homogenous which may be contributed to by the incompatibility of the materials. As for Figure 7d we observe a relatively smooth fractured surface showing the miscible nature of EPO(3) with PLA. This indicates that EPO(3) domains are uniformly distributed in the PLA phase, and distribution of EPO(3) component is not noticeable. The SEM micrograph of PLA/1 wt% EPO(1) and PLA/1 wt% EPO(3) displays a good adhesion between the components with a diffused polymer-plasticizer interface, producing single phase morphology [26,27]. This is indirectly reflected in more efficient load transfer under stress conditions, which was consistent with the improved tensile and impact properties of the blends.

Comparatively, SEM analysis of fracture surfaces of PLA/5 wt% EPO blends for EPO(1), EPO(2) and EPO(3) displayed similar micrographs as Figures 7e,f. These micrographs revealed poor interfacial adhesion properties, and the cavitation caused by debonding can be clearly identified. Presence of empty microvoids was observed in the blends indicating a formation of EPO rich phase in PLA matrix. EPO was located inside of the empty voids of PLA continuous phase. In addition, the stretched PLA phase in the blend may indicate ductile fracture of the PLA in the presence of EPO. Therefore, the degree of dispersion of the plasticizer in the PLA matrix is better at lower EPO loading, and the tendency to form empty voids and phase separation increases when the EPO content rises. These results are in good accordance with the mechanical property data, where the good interfacial adhesion results in higher mechanical strength of PLA/1 wt% EPO compared to PLA/5 wt% EPO blends [9].

### 2.5. Dynamic Mechanical Analysis of PLA/EPO Blends

**Figure 8:**
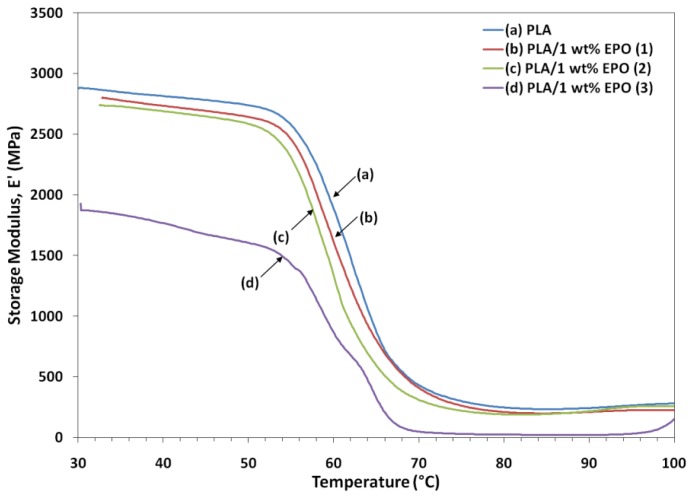
Storage modulus of neat PLA and PLA/1 wt% EPO blends.

**Figure 9:**
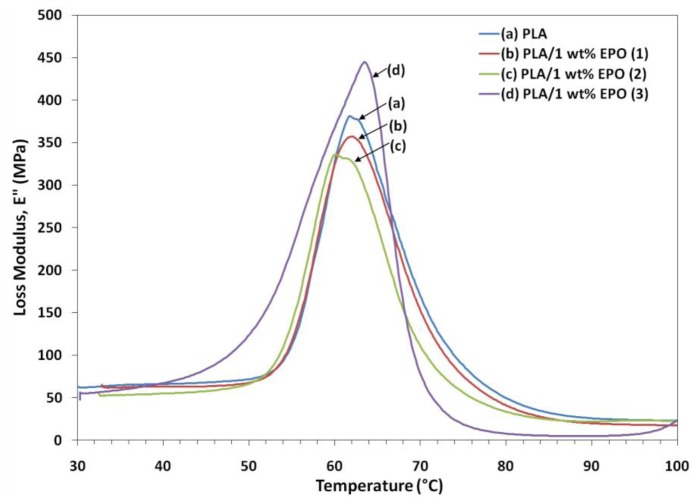
Loss modulus of neat PLA and PLA/1 wt% EPO blends.

**Figure 10:**
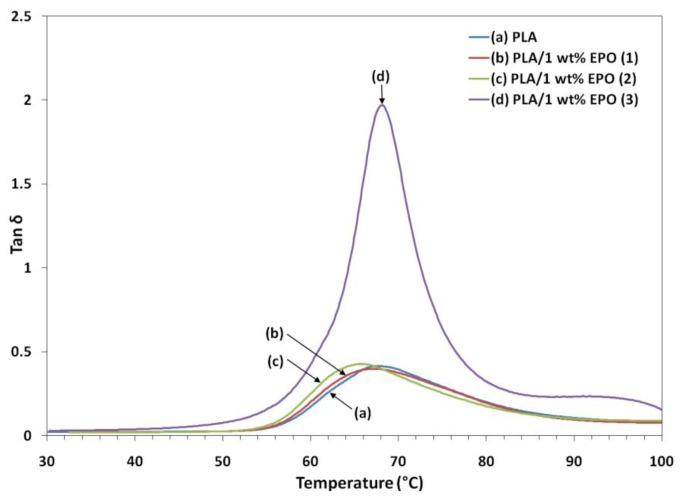
Loss factor of neat PLA and PLA/1 wt% EPO blends.

DMA is one of the most commonly used methods to obtain information related to the miscibility and phase behavior of polymer blends. DMA analysis investigates the thermomechanical properties of a given material and the setup allows us to obtain measurements of the storage and loss modulus (E′ and E″) and the loss factor (tan δ). Figures 8, 9 and 10 illustrate the dynamic storage modulus, loss modulus and tangent delta of PLA and PLA/1 wt% EPO blends, as a function of temperature, respectively.

The E′ values of neat PLA were slightly higher suggesting the high brittleness of the PLA than the blends. The lower storage modulus of the PLA/1 wt% EPO blends compared to neat PLA can be seen in Figure 8, indicating an increase in the flexibility of PLA imparted by the EPO. Further, there was a large drop on the storage modulus around 50–70 °C corresponding to the glass transition region, and there then followed a horizontal plane. EPO provides moderate toughening and elastomeric effect, which brings about a small decrease in the modulus values. Reduction in storage modulus with respect to temperature is related to softening of the matrix at higher temperature. As the temperature exceeds the softening point, mobility of matrix chains increase leading to sharp decrease of modulus at temperature between 50–70 °C [28].

The peak intensity of loss modulus represents the melt viscosity of a polymer. As observed, the PLA/1 wt% EPO(3) blend showed enhancement in loss modulus compared to neat PLA. This suggests an increase in the melt viscosity of the corresponding blend. However, a decrease in the loss modulus is observed for PLA/1 wt% EPO(1) and PLA/1 wt% EPO(2) blends signifies that the incorporation of EPO into PLA matrix decreases the melt viscosity of the corresponding blends. This is attributable to the addition of 1 wt% EPO(2) which decreases the flowability due to poor interaction and compatibility, leading to a less rigid polymeric material [17,29]. Besides, the fall in the modulus is attributed to an energy dissipation phenomenon involving cooperative motions of the polymer chains.

According to Liu *et al.* [28], the tan δ curve usually indicates the relaxation processes of polymers. The major relaxation process is associated with the glass-rubber transition (T_g_) of PLA. The height of the tan δ peak point out to the degree of crystallinity. Furthermore, the height of the tan δ peak is associated with the mobility of the amorphous region in the polymer blend. As presented, PLA/1 wt% EPO(1) and PLA/1 wt% EPO(2) blends display a reduction in the sharpness and height of tan δ peak, as the dispersed crystalline regions hinder the chain mobility in the amorphous regions. In addition, the restriction force increases directly with an increase in the crystallinity of the polymer. With the presence of 1 wt% EPO(1) and EPO(2), the molecular mobility of the materials decreases and the mechanical loss to reduce the intermolecular chain friction [30]. Comparatively, PLA/1 wt% EPO(3) blend exhibits a sharp and intense tan δ peak as there is no restriction to the motion of the main chain. This denotes that this blend contains a more amorphous region. This indicates that the blend has a good structural damping property, therefore, there is improved capacity to absorb mechanical energy by this blend compared to neat PLA, resulting in higher impact strength [31].

The T_g_ were obtained from the peak values of the tan δ curves. The T_g_ of PLA/1 wt% EPO blends of EPO(1), EPO(2), EPO(3) and neat PLA are 67.3, 65.8, 68.1 and 67.9 °C respectively. The slight shift in the T_g_ is a result of the plasticization effect. Furthermore, the molecular mobility of the PLA/EPO blend material is affected slightly with the addition of 1 wt% EPO.

### 2.6. Thermogravimetric Study of PLA/EPO Blends

**Figure 11:**
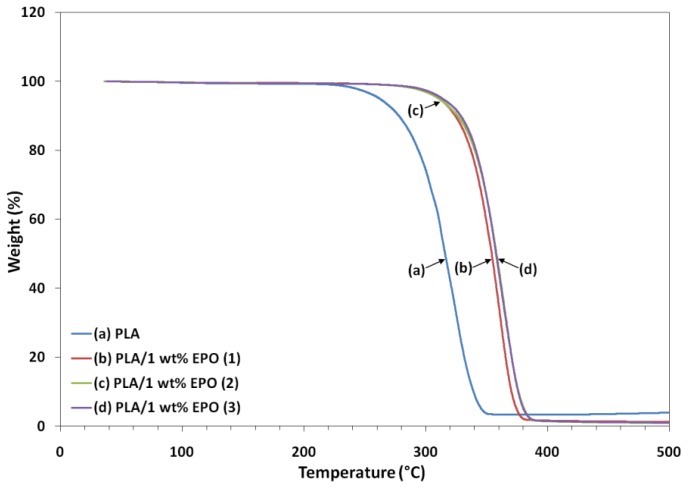
Thermogravimetric analysis (TGA) thermograms of neat PLA and PLA/1 wt% EPO blends.

**Figure 12:**
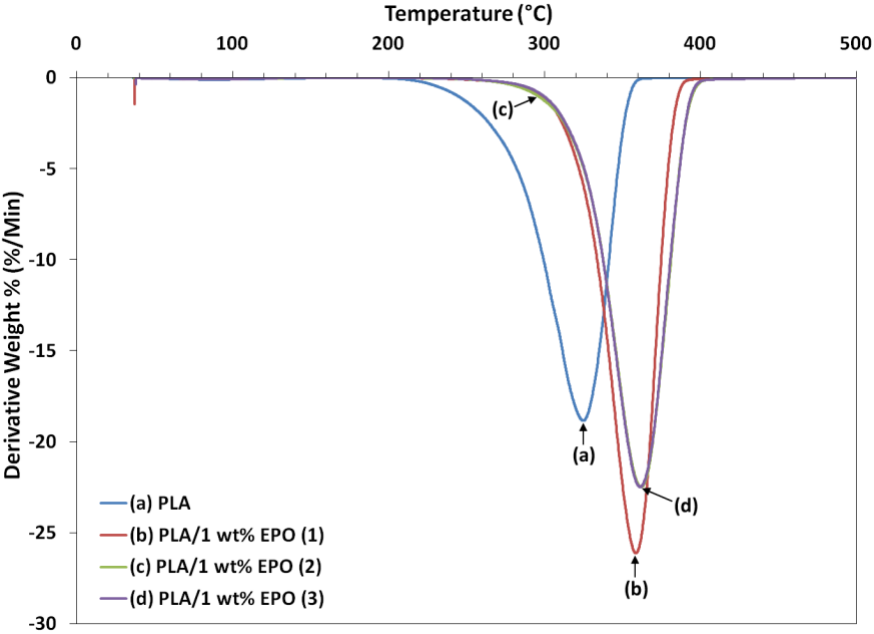
Derivative thermograms (DTG) of neat PLA and PLA/1 wt% EPO blends.

Thermogravimetric analysis (TGA) gives information on the structure of the intercalating molecules by the weight loss steps. Figures 11 and 12 show the TGA curves and derivative thermograms (DTG) of neat PLA and PLA/1 wt% EPO blends respectively. The characteristic thermal parameters selected were onset temperature, which is the initial weight loss temperature, and maximum degradation temperature, which is the highest thermal degradation rate temperature obtained from the peak of DTG thermograms [32].

Thermal degradation of neat PLA and PLA/1 wt% EPO blends takes place in a single weight loss step, which can be evidence from the DTG curves. The incorporation of EPO in PLA matrix has significantly affected the thermal degradation temperature by improving the thermal stability of the blends. From the characteristic temperatures in TGA curves, it can be seen that the PLA/1 wt% EPO blends appear to be thermally stable at temperatures lower than 270 °C. These materials lose about 10% of their weight at temperature between 270 and 330 °C, followed by an abrupt weight loss after 330 °C. Greater than 98% weight loss is observed for the three PLA/EPO blends, which were due to the thermal decomposition of the PLA polymer chains.

Neat PLA exhibits an onset temperature of 211.9 °C, which increased to 272.3, 270.4 and 279.3 °C when 1 wt% of EPO(1), EPO(2) and EPO(3) respectively incorporated into the blend. For PLA/1 wt% EPO(2) and PLA/1 wt% EPO(3), the difference in the curves can be hardly distinguished. However, a delay of the degradation is observed for PLA/1 wt% EPO(3) from the onset temperature. The blends showed a higher degradation temperature by approximately 27%, than neat PLA. The maximum degradation temperature (T_max_) also increased by compounding the PLA matrix with EPO. The T_max_ of the blends is higher, that is 358.6, 361.5, and 361.3 °C, for PLA containing 1 wt% of EPO(1), EPO(2) and EPO(3) respectively, compared to the PLA blend (324.8 °C).

These higher degradation temperatures may be attributed by the increase in molecular weight due to interaction between PLA matrix and EPO, or molecular chain-extension of the PLA matrix itself. Besides that, the presence of EPO dispersed homogeneously in the PLA polymer acts as a barrier sheet to prevent oxidation, as well as, hinders the permeability of volatile degradation products out from the blend materials and helps delay the thermal degradation process [33].

### 2.7. Differential Scanning Calorimetry Study of PLA/EPO Blends

**Figure 13:**
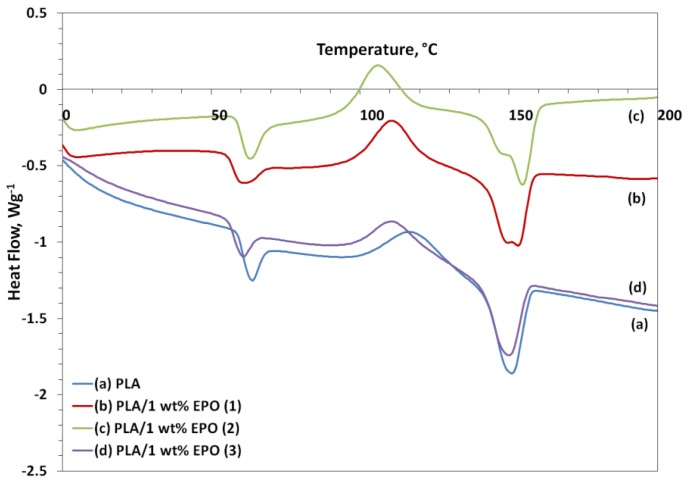
DSC thermograms of neat PLA and PLA/1 wt% EPO.

The DSC heating thermograms of neat PLA and plasticized PLA blends are illustrated in Figure 13. The DSC curves exhibits three thermal transitions, *i.e.*, glass transition (T_g_), crystallization (T_c_), and melting (T_m_) temperatures. Addition of EPO into PLA matrix decreased the T_g_ of neat PLA from 63.6 °C to 59.9 °C, 62.9 °C and 60.4 °C by adding 1 wt% of EPO(1), EPO(2) and EPO(3), respectively. The T_g_ of all three PLA/1 wt% EPO blends was lower than neat PLA due to the plasticization effect of EPO. In addition, the downward shift of T_g_ suggests some mixing of the EPO soft segments into the hard segment phase. This decrease in T_g_ can be explained on the basis of increased mobility of the soft segments because of the penetration of EPO units into the PLA hard segments [34].

With increasing EPO content to 5 wt%, T_g_ of PLA blends decreased slightly (not shown here) to 56.8 °C, 60.2 °C and 60.2 °C for PLA blends with 5 wt% of EPO(1), EPO(2) and EPO(3) respectively. As expected, increasing the EPO soft segment content has leaded to a slight decrease in the T_g_ of the blends due to the hard segments motion imposed by the EPO soft segments [34]. This also indicates that the PLA blends are a partially compatible polymer blend. However, the T_g_ with 5 wt% EPO was not considered, as the compatibility of blends became poor due to phase separation which can be evident from the SEM micrographs. Thus, the T_g_ at 1 wt% EPO was considered as the minimum T_g_ as the PLA blends have greatly enhanced properties in terms of tensile strength and impact strength.

However the T_g_ obtained from DSC and DMA does not correlate. The T_g_ of PLA/1 wt% EPO blend of EPO(1), EPO(2) and EPO(3) obtained from DMA analysis are 67.3 °C, 65.8 °C and 68.1 °C, respectively. While T_g_ of neat PLA from DMA measurements were 67.9 °C. The difference in the T_g_ values based on DSC and DMA can be explained by the different measuring principles and heating rates selected [35]. The neat PLA indicated a melting endothermic peak at 151 °C. As observed from Figure 12, the DSC thermogram of PLA/1 wt% EPO(1) and PLA/1 wt% EPO(2) exhibits two distinct peaks of melting temperature at 149.8 °C and 153.1 °C, as well as, 148.5 °C and 155.1 °C respectively. According to Hala *et al.* [34], the lower melting endotherm corresponds to the crystalline phase of PLA/EPO soft segments and the higher one to the crystalline phase of hard segments. However, there is possibility that these blend systems are not completely miscible [36]. Conversely, in the heating scan of PLA/1 wt% EPO(3) only one T_m_ peak appeared at 150.1 °C resembling to the crystalline phase of soft segments. The downward shift in T_m_ of this blended film indicates the increased miscibility of the components in the blends [37].

Furthermore, T_c_ was detected for neat PLA and PLA/1 wt% EPO blends of EPO(1), EPO(2) and EPO(3) at 117.9 °C, 110.8 °C, 105.9 °C and 111.1 °C. The incorporation of EPO dispersed in PLA leads to a significant shift of the crystallization peak towards lower temperatures. The T_c_ decreased because PLA chains possessed higher mobility after the addition of EPO, which made it easier for them to fold into a crystalline lattice [38].

When we discuss the mechanical properties of PLA blends in connection with the degree of incorporation, the influence of degree of crystallinity should also be considered because it may change with the addition of EPO. The relative percent crystallinity (X_c_%) of EPO segments in PLA was calculated from the enthalpy obtained from the DSC curves. The percentage of crystallinity, X_c_ were calculated using the following equation:

(1) $${\text{X}}_{\text{c}}\;(\%)=\frac{|\Delta {\text{H}}_{\text{c}}+\Delta {\text{H}}_{\text{m}}|}{|\Delta {\text{H}}_{\text{m}}\;(100\%)|}\times 100\%$$

here, Delta;H_c_ is the heat of crystallization (J/g), Delta;H_m_ is the heat of fusion (J/g), and Delta;H_m_ (100%) is the enthalpy of melting of 100% crystalline PLA, equals to 93 J/g [39]. In this investigation, the neat PLA showed X_c_ of 69.0%, which means that PLA is semi-crystalline. The X_c_ of PLA/1 wt% EPO(1) and PLA/1 wt% EPO(2) increased to 72.3% and 71.8% respectively. These results clearly indicate that PLA/1 wt% EPO(1) and PLA/1 wt% EPO(2) are identical in terms of crystallinity, suggesting that these two polymers were previously subjected to the identical processing conditions (thermal history). PLA/1 wt% EPO(3), on the other hand, has a sharper melt and lower crystallinity of 58.3% indicating different processing conditions and different end-use properties [40]. Besides that, high plasticizer content contributes to a decrease in the crystallization peak as a result of phase separation.

### 2.8. X-Ray Diffraction (XRD) Study of PLA/EPO Blends

**Figure 14:**
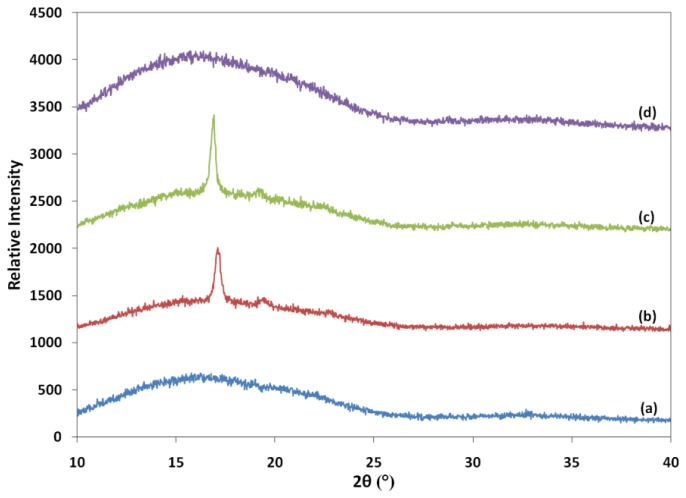
X-Ray Diffraction (XRD) pattern of (**a**) neat PLA; (**b**) PLA/1 wt% EPO(1); (**c**) PLA/1 wt% EPO(2); and (**d**) PLA/1 wt% EPO(3) blends.

XRD patterns of neat PLA and PLA/1 wt% EPO blends are shown in Figure 14. The XRD patterns for PLA exhibit a broad diffraction peak centered at 2θ ≈ 16°. The PLA did not show any characteristic peak, which indicates that the structure is amorphous. The neat PLA employed in this research has 5.49 Å of interlayer spacing at 2θ = 16.22°.

The XRD pattern of PLA/1 wt% EPO(1) and PLA/1 wt% EPO(2) blends has a very strong crystalline peak at 2θ of 17.09° and 16.85°, which correspond to the basal spacing 5.18 Å and 5.26 Å, respectively. The shift to a higher angle indicates a decrease in the corresponding interlayer spacing, which is contributed to by the fact that the blend components have an ordered structure. As the PLA chain was the main component of the blend, the position of crystalline peak was almost similar to that of the PLA.

Unlike PLA/1 wt% EPO(1) and PLA/1 wt% EPO(2) blends, PLA/1 wt% EPO(3) blend shows absence of crystalline peak, indicating an amorphous structure. This blend reported an increment of interlayer spacing, where the *d*-spacing increased from 5.49 Å (neat PLA) to 5.79 Å at diffraction peak centered at 15.28°. The increment in the interlayer spacing of PLA evidences that this blend has more amorphous region and the addition of 1 wt% EPO(3) creates a less-ordered structure, thus crystallization becomes more difficult [41]. Interestingly, these results contradict the percent crystallinity calculated from DSC data, which revealed that PLA and PLA/1 wt% EPO(3) are semi-crystalline instead of amorphous observed from the XRD analysis. This results from the different conditions of instrumental analysis and the distribution of amorphous and crystalline region in the semi-crystalline blends, which affects the crystallinity data obtained.

## 3. Experimental

### 3.1. Materials

Poly(lactic acid) pellets, commercial grade 4042D, were purchased from NatureWorks^TM^ LLC (United States) with 1.24 g/cm^3^ as density. The polymer was dried for at least 3 h at 50 °C before being used in the blend preparation. Three different samples of epoxidized palm oil (EPO) were supplied by Advanced Oleochemical Technology Division (AOTD), Malaysian Palm Oil Board (MPOB). The characteristics of these EPO obtained are listed in Table 1.

### 3.2. Preparation of PLA/EPO Blends

The PLA/EPO blends were produced by melt blending technique using a twin counter-rotating mixer (Brabender internal mixer, MELCHERS, Germany) at 170 °C for 15 min and rotor speed of 50 rpm. The weight ratios of PLA/EPO(1) were 100/0, 99/1, 98/2, 97/3, 96/4, and 95/5. After melt blending, the sheets were prepared using a hydraulic hot-press (Hsin-Chi Machinery Company Ltd., Taiwan) with a pressure of 110 kg/cm^3^ at 160 °C for 10 min, and then, cooled to room temperature to produce 1.00 mm and 3.00 mm uniform thickness sheets. The same procedure were repeated for the preparation of PLA/EPO(2) and PLA/EPO(3) blends. Subsequently, the blend sheets were characterized for mechanical, thermal and morphological properties.

### 3.3. Tensile Test

Tensile test was carried out using Instron Universal Testing Machine (Model 4302 Series IX) based on ASTM D638. Seven dumbbell shape specimens were prepared from each composition. The average thickness and average width of the gauge section of each specimen were calculated. The test was conducted at a constant crosshead speed of 5 mm/min, load cell of 1 kN and a gauge length of 10 mm. Tensile strength, tensile modulus and elongation at break were obtained using Instron Series IX software. An average of five results was taken as the resultant value.

### 3.4. Flexural Test

Flexural test was conducted in accordance with ASTM D790, using Instron Universal Testing Machine (Model 4302 Series IX) equipped with a 1 kN load cell. Seven specimens in rectangular shape with the dimension 127.00 mm × 12.70 mm × 3.00 mm of size were tested for each composition. Flexural strength and flexural modulus were obtained at constant crosshead speed of 3 mm/min. An average of five results was taken as the resultant value.

### 3.5. Izod Impact Test

Impact test was conducted using Izod Impact Tester (International Equipments, India) with a 453 g (1.0 lb) pendulum according to ASTM D256. Five specimens with length 63.50 mm, 12.70 mm width and thickness approximately 3.00 mm, were tested for each composition. The impact strength (J/m) was calculated by dividing the energy obtained (J) with the thickness of specimen (m). An average of three results was taken as the resultant impact strength.

### 3.6. Scanning Electron Microscopy (SEM)

Morphology analysis of tensile fractures surface of blends was observed by Scanning Electron Microscope (Model Philips XL 30), with an acceleration voltage of 20 kV. In order to avoid electrostatic charging during electron irradiation, the specimens were sputter coated with gold a few nanometres in thickness, in vacuum conditions prior to each examination.

### 3.7. Dynamic Mechanical Analysis (DMA)

Thermal dynamic analysis (DMA) was performed according to ASTM D5023 on a dynamic mechanical analyzer (Perkin-Elmer PYRIS Diamond DMA), using bending mode. The temperature scan was from ambient temperature (25 °C) to 100 °C at a constant heating rate of 2 °C/min and the frequency of dynamic force of 1 Hz, under nitrogen atmosphere. The storage modulus (E′), loss modulus (E″), and loss factor (tan δ) of each specimen were obtained as a function of temperature.

### 3.8. Thermogravimetric Analysis (TGA)

The thermal stability of the samples was studied by using the Perkin Elmer TGA7 Thermogravimetric Analyzer. The weight of the samples used was approximately 10.0 mg and were heated from 25 °C to 500 °C at the heating rate of 10 °C/min. The analysis was carried out in nitrogen atmosphere with nitrogen flow rate of 20 mL/min. The weight loss of samples were recorded and plotted as a function of temperature.

### 3.9. Differential Scanning Calorimetry (DSC)

The melting and crystallization behavior testing of the blends was performed using a DSC analyzer machine, Mettler Toledo. The scan was carried out at the rate of 10 °C/min from 0 °C to 200 °C with a flow rate of nitrogen gas of 50 mL/min. The method utilizes differential heat flow and temperature, normally associated with transition in materials.

### 3.10. X-Ray Diffraction (XRD) Analysis

X-ray diffraction measurement was carried out by using a Shimadzu XRD 600 X-ray diffractometer with CuKα radiation (λ = 1.542 Å) operated at 30 kV and 30 mA. Data were collected within the range of scattering angles (2θ) of 10° to 40° at the rate of 2°/min. The basal spacing was derived from the peak position (*d*_001_ reflection) in the XRD diffractogram according to the Bragg equation (λ = 2*d* sinθ).

## 4. Conclusions

The effect of different types of EPO and its loading were studied on PLA/EPO blends. The optimum EPO loading with enhanced mechanical and thermal properties of PLA is 1 wt%. PLA/1 wt% EPO(3) blend showed excellent properties compared to PLA/1 wt% EPO(1), PLA/1 wt% EPO(2) and neat PLA. The tensile strength, flexural strength, impact strength and elongation at break of PLA/1 wt% EPO(3) were improved by 13%, 25%, 23% and 77% respectively compared to neat PLA. This reveals good stress transfer of the material. Addition of 1 wt% EPO(3) also significantly decreased the storage modulus while increased the loss modulus and loss factor. From XRD patterns, it can be inferred that the amorphous phase of the PLA/1 wt% EPO(3) blend was increased, whereas the crystalline phase increased for PLA/1 wt% EPO(1) and PLA/1 wt% EPO(2), compared to neat PLA. From the TGA results, there is a significant improvement about 27% on the thermal stability of blends with the addition of EPO. Furthermore, the DSC results illustrate a decrease in the glass transition temperature due to the plasticizing effect of EPO. The tensile fractured surface morphology of the PLA/1 wt% EPO(3) shows a smooth surface without entanglement and aggregation, indicating good interaction between PLA and EPO(3). This is in agreement with the significant improvement of mechanical properties. Thus, this blend can be considered as an alternative to the conventional plastics used. Additionally, EPO can be seen as a potential useful plasticizer.

## Figures and Tables

**Table 1:** Properties of EPO.

Sample Name	EPO(1)	EPO(2)	EPO(3)
**Sample Composition**	Epoxidized palm olein	Epoxidized palm kernel oil with small amount of soybean oil	Epoxidized palm olein with small amount of soybean oil
**Oxygen Oxirane Content (%)**	3.2309	1.750	3.5803
**Acid Value (mg KOH/g sample)**	0.4287	0.1737	0.5990
**Iodine Value (g I_2_/100 g sample)**	0.6371	3.5674	0.4999
**Moisture Content**	0.08	0.03	0.06
**pH (pH test paper)**	5–6	5–6	5–6
